# Growth characteristics of the novel goose parvovirus SD15 strain in vitro

**DOI:** 10.1186/s12917-019-1807-y

**Published:** 2019-02-19

**Authors:** Jinyue Zhang, Peng Liu, Yuanyuan Wu, Mingshu Wang, Renyong Jia, Dekang Zhu, Mafeng Liu, Qiao Yang, Ying Wu, Xinxin Zhao, Shaqiu Zhang, Yunya Liu, Ling Zhang, Yanling Yu, Yu You, Shun Chen, Anchun Cheng

**Affiliations:** 1 Institute of Preventive Veterinary Medicine, Sichuan Agricultural University, No. 211 Huimin Road, Wenjiang District, Chengdu city, 611130 Sichuan province China; 2 Research Center of Avian Disease, College of Veterinary Medicine, Sichuan Agricultural University, Chengdu, 611130 Sichuan China; 3 Key Laboratory of Animal Disease and Human Health of Sichuan Province, Sichuan Agricultural University, Chengdu, 611130 Sichuan China

**Keywords:** Novel goose parvovirus (NGPV), Proliferation, Growth curve, Apoptosis

## Abstract

**Background:**

Short beak and dwarfism syndrome (SBDS) was caused by novel goose parvovirus (NGPV)--a variant of goose parvovirus (GPV). Ducks infected with NGPV shows clinical signs including growth retardation and protrusion of the tongue from an atrophied beak. SBDS outbreak was first reported at the northern coastal provinces of China during 2015 and it was again reported in Sichuan, an inland province of China in 2016. The disease caused a huge economic loss in Chinese duck feeding industry.

**Results:**

The SD15 strain of NGPV was isolated from liver and intestinal tract tissue samples of infected ducks. Real-time quantitative PCR (qPCR) was used to estimate viral load in embryonated eggs and cells infected with adapted virus. The data showed that duck embryo fibroblasts (DEFs) were permissive to NGPV, while goose embryo fibroblasts (GEFs) cells were not, and the copy numbers of SD15 in the allantoic fluid of infected eggs remained at 10^5.0^–10^6.5^ copies/ml. The adaption procession of the virus was determined via qPCR, and viral proliferation was detected through indirect fluorescent antibody assay (IFA) in DEFs. It was further determined that viral copy numbers peaked at 96 h post-inoculation (hpi), which is the best time to harvest the virus in DEFs. Cytotoxic effects and cell death were observed at 72 hpi in SD15 infected DEFs, yet SD15 did not induce apoptosis.

**Conclusions:**

The growth characteristics of SD15 strain of NGPV determined would be beneficial for further molecular characterization of these viruses and develop potential vaccines if required.

## Background

Short beak and dwarfism syndrome (SBDS) was first reported in Mule ducks in France during 1971–1972 [1], then in Taiwan during 1989–1990 [2]. In 2015, SBDS was reported in the northern coastal provinces of China, including Shandong, Jiangsu, Hebei and Anhui [3, 4]. A year later, the disease was reported in Sichuan, an inland province of China [5]. The etiological agent of SBDS is a novel goose parvovirus (NGPV), which shared the highest homology with goose parvovirus (GPV) and was regarded as a variant of GPV [1, 4]. NGPV was not a lethal etiological agent in ducks, but farmers suffered great economic loss due to reduced sales of NGPV-infected ducks. The virus currently spread from the coast to the inland regions of China [5, 6]. Waterfowl parvovirus infections are generally divided into goose parvovirus (GPV) and Muscovy duck parvovirus (MDPV). GPV infects both goslings and Muscovy ducks, causing Derzsy’s disease [7], while MDPV infects Muscovy ducklings, causing a disease known as “three-week disease” in China. Both types of infections are fatal to waterfowls. NGPV infects 1-day-old Cherry ducks and Mule ducks and demonstrates a high rate of infection with low mortality. Most ducks develop asymptomatic infections, but those with significant clinical symptoms showed growth retardation, atrophied beaks, a protruding tongue outside of the beak, swelling and hemorrhage of the thymus, and fractured feathers and legs [1, 3, 8].

NGPV, a waterfowl parvovirus, along with GPV and MDPV, belongs to *Anseriform dependoparvovirus 1* [9, 10]*.* In the family Parvoviridae, viruses contain a linear, single-stranded DNA genome of approximately 5 kb in length [10]. The full-length genome contains two open reading frames (ORFs): the left ORF codes for the non-structural proteins NS1 and NS2 [11], and the right ORF codes for the structural proteins VP1, VP2 and VP3 [12]. Additionally, there are two inverted terminal repeats (ITRs) at both ends of the genome [12, 13].

Waterfowl parvovirus is classified as a dependovirus based on its genomic sequence but is distinguished from other dependoviruses by its ability to replicate autonomously in vitro without helper-viruses [14, 15]. During propagating, waterfowl parvovirus shows the characteristics of both autonomous and dependovirus parvoviruses [5, 16]. Certain parvoviruses such as H-1 can cause cytopathic effects (CPE) and induce apoptosis in infected cells through the process of replication [17], but it is unclear whether NGPV has this apoptosis effect.

As a waterfowl parvovirus, NGPV can propagate autonomously. In previous reports, the viruses isolated by Palya were adapted in Muscovy duck eggs after being blindly propagated for one or two passages [1]. The other three strains, SDLC01, DS15 and M15, were adapted in ducks after three or four blind propagations. Strains SDLC01 and M15 could only cause CPE in duck embryo fibroblasts (DEFs) [4, 8, 9]. Collectively, it was demonstrated that NGPV could replicate in duck eggs and DEFs, but the details about the adaption process and growth curve of NGPV were not reported. In this study, the NGPV strain SD15 was isolated, and its growth characteristics were investigated, laying a foundation for further vaccine and viral molecular research.

## Methods

### Samples collection

Liver and intestinal tract tissue samples were collected from two dead SBDS ducks from Shandong province. The ducks were identified as NGPV positive infection. The samples were homogenized in phosphate buffered saline (PBS, pH 7.2) and centrifuged at 6000 rpm for 15 min. The supernatant was filtered through a 0.22-μm filter. And then the filtered supernatant was stored at − 80 °C.

### Virus isolation and adaption in duck embryos

All the embryos used in the study were 9-day-old and purchased from the breeding center of Sichuan Agricultural University (Ya’an, Sichuan province). The duck embryos were inoculated with the filtered supernatant (0.2 ml/embryo) prepared before and monitored daily for 7 days and allantoic fluid were collected and saved at − 80 °C for the next infection and detecting copy numbers. The experiment has been approved by Animal Ethics Committee of Sichuan Agricultural University, and was performed according to the experiment operational guideline of Sichuan province, China. Before break-out, the duck embryos were placed in a 4 °C environment for 4 h.

The virus was continually passaged for 12 times in duck embryos (*n* = 3 embryos for each passage). The copy numbers of viruses were estimated for every two passage using qPCR.

### Virus isolation and adaption in DEFs and GEFs

The DEFs and GEFs were primary cells separated from duck and goose embryos, respectively. DEFs and GEFs were inoculated with filtered liquid at a 1:10 dilution. At the same time, the DEFs and GEFs in the control group were inoculated with PBS. Seven days after inoculation, the infected cells underwent three freeze-thaw cycles prior to harvest and storage at − 80 °C for the next infection and detecting copy numbers. The virus was then serially passaged at least 5 times in DEFs or GEFs. After the virus adapted to the cells, the CPE was observed daily for up to 7 days, and viral copy numbers were detected via real-time quantitative PCR (qPCR) every two passage.

### Estimation of the copy numbers of NGPV

qPCR was used to detect the copy numbers of NGPV. 200 μl liquid of each sample collected before was used to extract DNA. And viral DNA was extracted using the TIANamp Virus DNA/RNA Kit (TIANGEN, Beijing, China) according to the manufacturer’s protocol. The primers were designed using Primer Premier 5.0 as follows: forward, 5’-TGCCGATGGAGTGGGTAAT; reverse, 5’-CGCCAGGAAGTGCTTTAT. These primers amplified a sequence based on the NGPV strain SDCL01 (GenBank: KT343253). qPCR was performed by Bio-Rad CFX96 Real-Time Detection System (Bio-Rad, CA, USA). The system contained 5 μl of DNA polymerase (Innovagene, Hunan, China), 0.3 μl of 10 μM forward and reverse primers each, 0.4 μl of DNA template, and 4 μl of ddH_2_O. The reaction was carried out using the following PCR program: pre-denature at 95 °C for 10 min; denature at 95 °C, anneal at 59.4 °C; and extend at 72 °C for 30 s.

### Indirect fluorescent antibody assay (IFA)

Indirect immunofluorescence assay (IFA) was used to investigate the growth cycle of NGPV in DEFs. NGPV-infected DEFs were collected at 24, 48, 72, 96 and 120 h post-inoculation (hpi) (*n* = 3 samples for each time point). And the cells in the control group were dealt with the same volume of PBS. Collected cells were fixed in 4% paraformaldehyde for 24 h at 4 °C, then permeabilized in 0.25% Triton X-100 for 1 h at 4 °C. The cells were then washed three times with PBS, blocked with 5% bovine serum albumin diluted by PBS for 1 h and then incubated with an NGPV polyclonal antibody as the primary antibody (mouse antiserum against the novel goose parvovirus was made by our lab previously, 1:100 diluted in PBS) at 37 °C for 1 h. Following incubation, the cells were washed again and incubated with a fluorescein isothiocyanate-labeled goat anti-mouse antibody (Ruiying Biological, China, 1:200 diluted in PBS). Cellular fluorescence was observed using a microscope (Nikon, Tokyo, Japan).

### Cytotoxicity assay

Cell counting Kit-8 (MenChem Express, NJ, USA) was used to test cytotoxic effects of SD15. Cells were seeded in 96-well plates and infected with NGPV. At 24, 48, 72, 96 and 120 hpi (*n* = 3 samples for each time point), 10 μl of CCK-8 solution was added into every well with cells (NGPV and MOCK-infected) and incubated for 1 h according to the manufacturer’s protocol. The microplate reader was used to measure the absorbance at 450 nm. Control experiments were carried out in parallel by incubating cells with PBS instead of virus.

### Apoptosis assay

PE Annexin V Apoptosis Detection Kit I (BD Bioscience, NJ, USA) was used with flow cytometry to determine if NGPV could induce apoptosis. DEFs were seeded in 6-well plates and infected with NGPV. And the cells in the control group were dealt with the same volume of PBS. The infected DEFs were harvested at 24, 48, 72, 96 and 120 hpi (*n* = 3 samples for each time point) and washed twice in cold PBS. The cells were suspended in 1 × Binding Buffer, and then PE Annexin-V and 7-Amino-Actinomycin (7-AAD) were added according to the manufacturer’s protocol. The cells were then incubated for 15 min at room temperature in the dark. A Beckman CytoFLEXflow cytometer (Beckman Coulter, CA, USA) was used to analyze the samples.

### Statistical analysis

GraphPad Prism v5.02 (GraphPad Software Inc., CA, USA) was used to analyze the data. And results were expressed as the mean ± SD and a significance threshold of *p*-value < 0.05. Differences between experimental groups were analyzed via Student’s t-test.

## Results

### Virus isolation and adaption

**Fig. 1:**
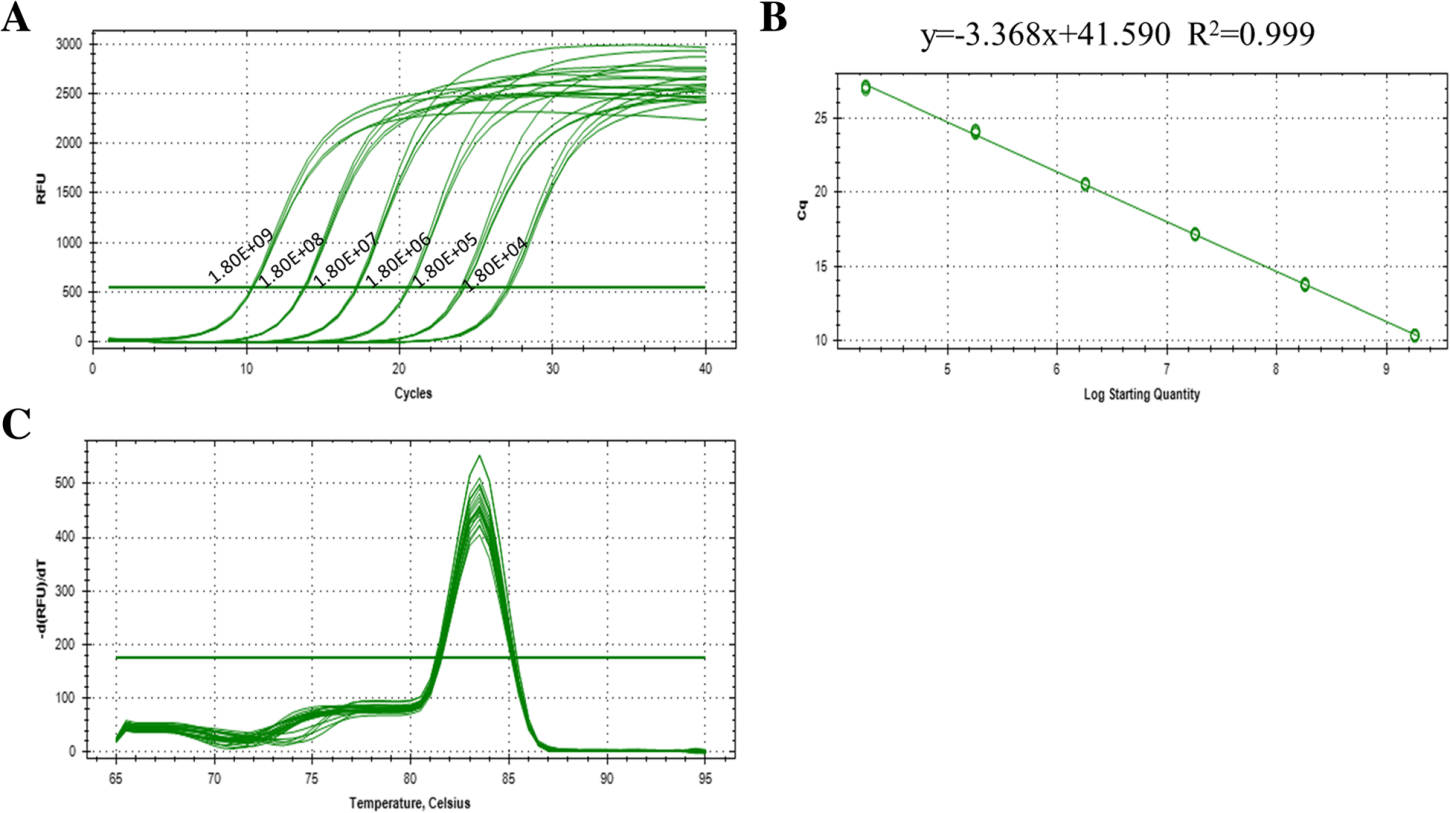
Performance of the real-time PCR assay. **a** Amplification curves of the standards by real-time PCR. **b** Standard curve of the real-time PCR. **c** Melting curves of the real-time PCR

NGPV was continually blind-passaged in duck embryos, DEFs, and GEFs. In order to detect the virus load, qPCR assay was built. The standard curve of it revealed the linear relationship between template concentration and Cq value: y = − 3.368x + 41.590. And the regression coefficient R^2^ = 0.999 and the melt curves indicated that the assay was highly reproducible and specificity and the method can be used to quantify the viral copy numbers (Fig. 1a-c).

**Fig. 2:**
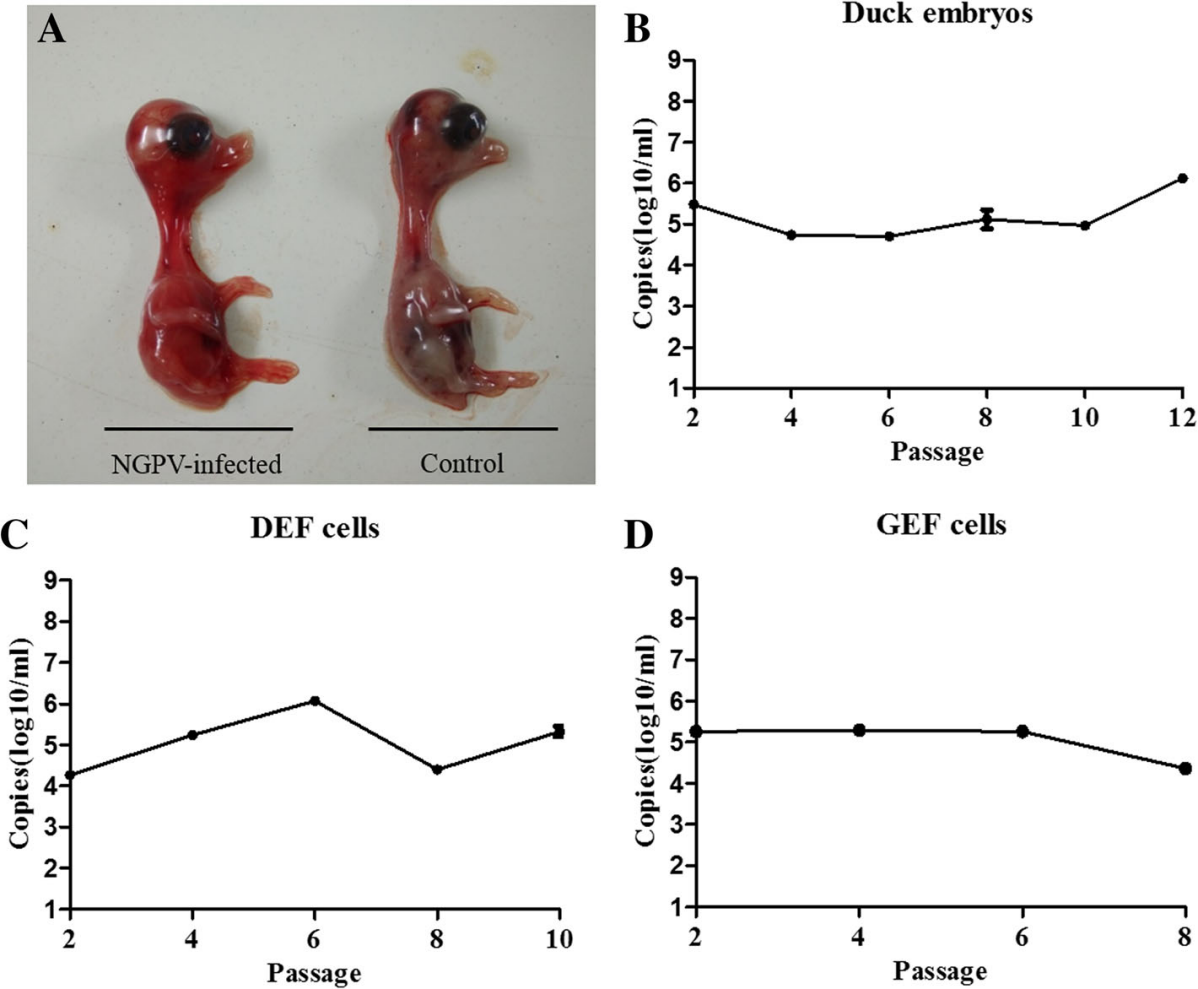
Adaption of NGPV in eggs and cells. **a** Picture of an NGPV-infected duck embryo (left) and a control duck embryo (right). **b** Copy numbers of NGPV in the allantoic fluids of infected duck embryonated eggs. **c** Copy numbers of NGPV in the infected DEF cells. (D) Copy numbers of NGPV in the infected GEF cells. Each sample was analyzed in triplicate

After blindly passaged four passages, 60% of duck embryos died at 60 hpi with systemic hemorrhaging (Fig. 2a), and the viral copy numbers of NGPV-infected duck embryos were stable at 10^5.0^–10^6.5^ copies/ml from the first to twelfth passage (Fig. 2b).

At the fourth passage in DEFs, CPE was observed and the viral copy numbers remained at 10^4.5^–10^6.0^ copies/ml (Fig. 2c). However, in NGPV-infected GEFs, no CPE could be observed after the tenth passage, and the copy numbers began to drop after the sixth passage (Fig. 2d).

### NGPV detection by IFA

**Fig. 3:**
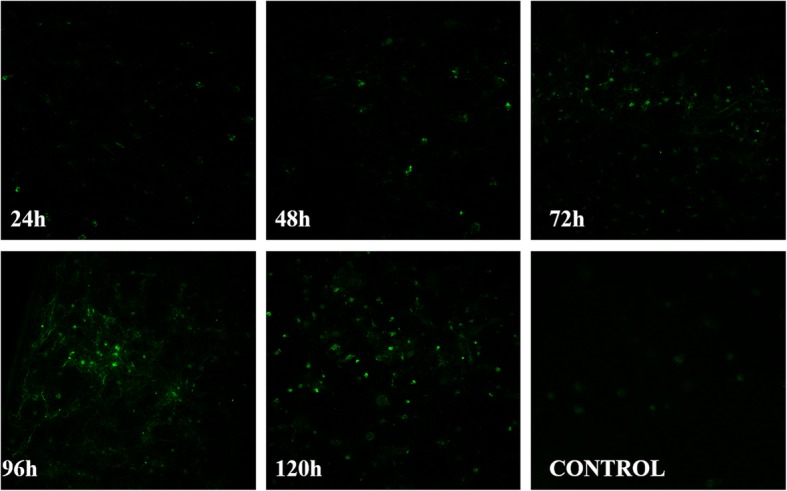
Immunofluorescent analysis of the NGPV growth curve in DEF cells. NGPV was detected at 24, 48, 72, 96 and 120 hpi by IFA. NGPV polyclonal antibody was used as the primary antibody (1:100 dilution), and fluorescein isothiocyanate-labeled goat anti-mouse was used as the secondary antibody (1:200 dilution)

IFA was used to quantify NGPV propagation in DEFs via green fluorescent intensity. From 24 to 48 hpi, low levels of green fluorescence were observed. At 48 hpi, the virus replicated rapidly and reached maximum fluorescent intensity at 96 hpi. At 120 hpi, the viral load plateaued and remained stable. No green fluorescent signal was observed in control groups (Fig. 3).

### Cytotoxic effect and CPE observation of NGPV infected DEF cells

**Fig. 4:**
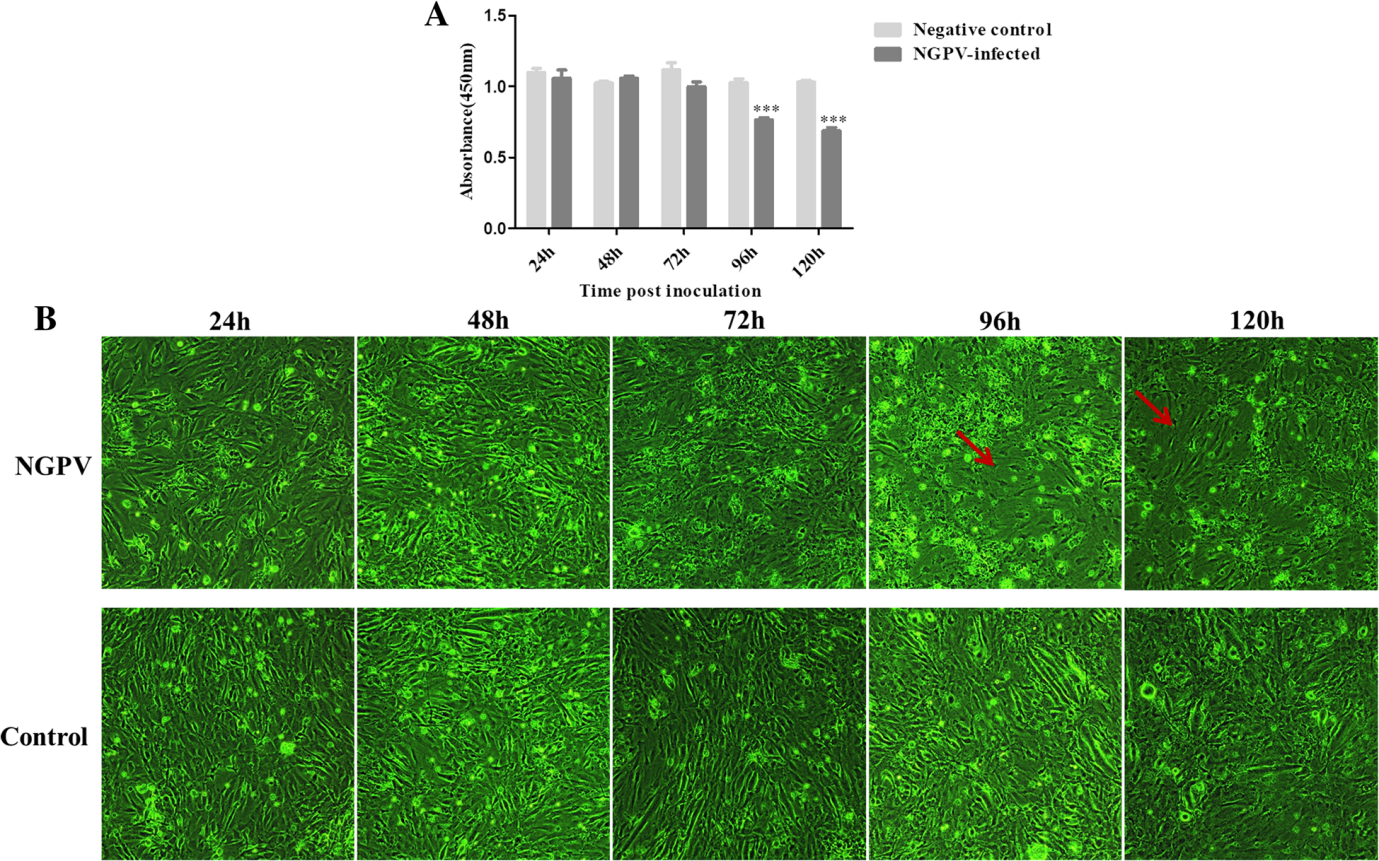
The cell cytotoxic effect and CPE caused by NGPV in infected DEF cells. All the cells were infected with NGPV, and then the cytotoxic effect (**a**) and CPE (**b**) were observed at 24, 48, 72, 96 and 120 hpi. Red arrows represent the CPE on DEFs. Each sample was analyzed in triplicate. Groups denoted by (***) indicate a significant difference at *P*<0.001

The CCK-8 assay was used to explore the cytotoxic effect of NGPV on DEFs. CPE was observed in NGPV infected DEFs at 96 and 120 hpi. As shown in Fig. 4a and b, no cytotoxic effect was detected during 24–72 hpi, and no CPE was detected in either NGPV or Mock-infected cells. However, at 96 and 120 hpi, a significant cytotoxic effect was observed. While 80% of cells died without showing obvious plaque, it was observed that cells became round, experienced lysis and fragmentation and developed irregular cell membranes between 72 and 120 hpi.

### The apoptosis assay of NGPV infected DEF cells

**Fig. 5:**
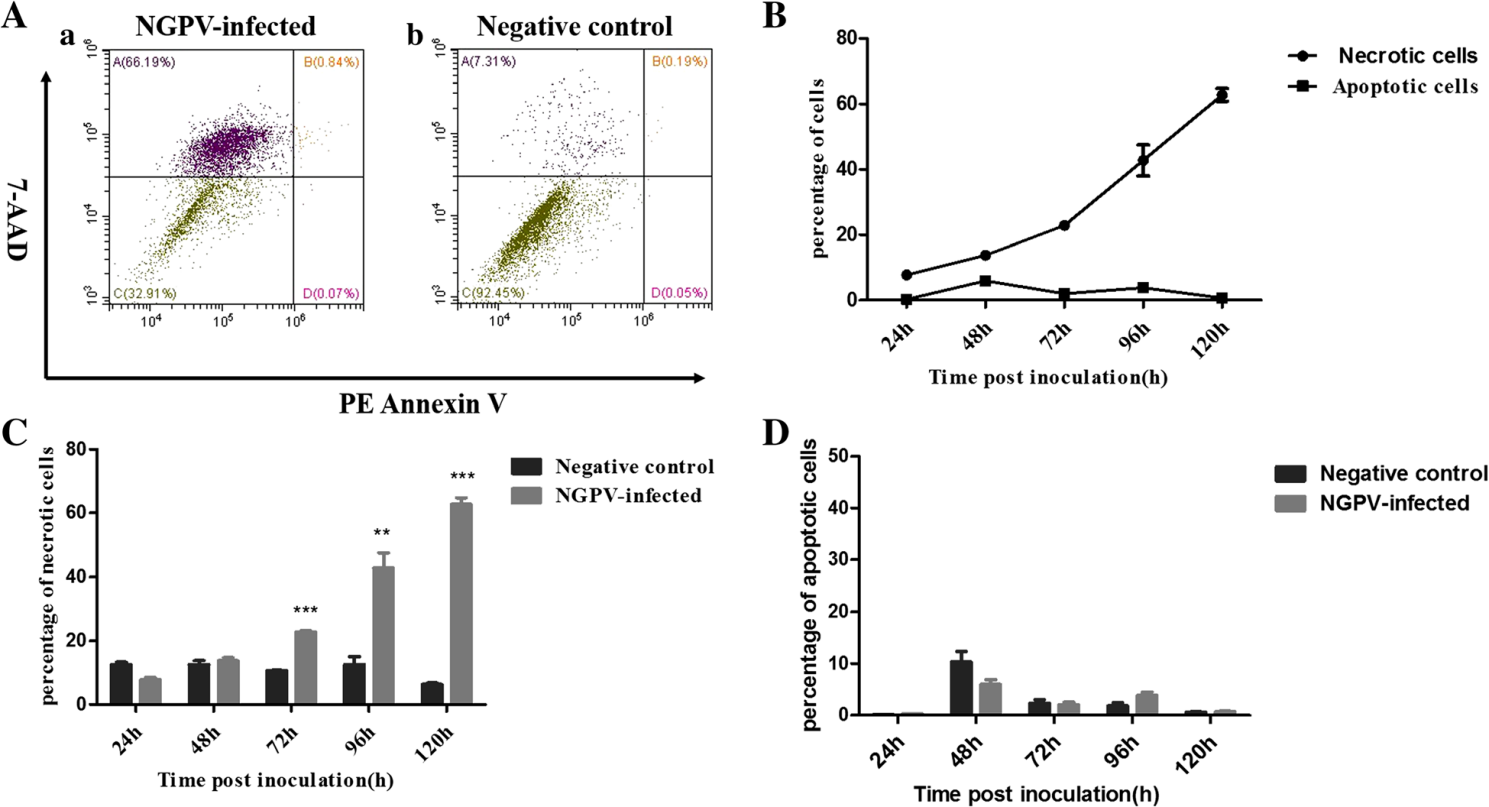
Flow cytometry analysis of NGPV infected DEF cells. **a** Annexin and V/7-AAD double staining was used to analysis the apoptosis in DEF cells. The DEF cells were harvested at 120 hpi. Compartment A: 7-AAD-positive; Compartment B: double-positive; Compartment C: double-negative; Compartment D: Annexin V-positive. (A-a) NGPV infected group and (A-b) Negative group. **b** The time course study of the percentage of necrotic and apoptotic NGPV infected cells. **c** The time course study of the percentage of necrotic cells. **d** The time course study of the percentage of apoptotic cells. Each sample was analyzed in triplicate. Groups denoted by (**) indicate a significant difference at *P*<0.01, and groups denoted by (***) indicate a significant difference at *P*<0.001

NGPV resulted in significant cytotoxic effects and CPE at 96 and 120 hpi, which led us to question whether NGPV could cause cell death by inducing apoptosis, similar to most other parvoviruses reported. DEFs were harvested and stained with PE Annexin V and 7-Amino-Actinomycin (7-AAD), and then the number of viable, apoptotic and necrotic cells were detected by flow cytometry. 7-AAD^−^/PE Annexin V^+^, 7-AAD^+^/PE Annexin V^+^ and 7-AAD^+^/PE Annexin V^−^ label early-stage apoptotic cells, late-stage apoptotic cells, and necrotic cells, respectively. Most cells died at 120 hpi (Fig. 5a). As shown in Fig. 5b, the percentage of necrotic cells increased slightly at 24–48 hpi, rose rapidly during 72–120 hpi and reached the peak at 120 hpi. The proportion of necrotic cells reached nearly 70% among all the cells including apoptotic, necrotic and viable cells at 120 hpi (Fig. 5c). However, the proportion of apoptotic cells, including early and late-stage apoptotic cells, remained nearly constant without significant change compared to the negative control group (Fig. 5b-d).

## Discussion

In 2015, the NGPV outbreak in a Northern China coastal city was the result of a novel goose parvovirus, which was closely related to the goose parvovirus (GPV) [4]. In our study, SD15 was isolated from liver and intestinal tract tissue obtained from infected ducks. After blind-propagation for 3 passages in vitro, it was found that SD15 infection of duck embryos resulted in death at 48–60 hpi with systemic hemorrhage. Further, viral copy numbers detected by qPCR remained stable at 10^5.0^–10^6.5^ copies/ml across 10 passages. In SD15-inoculated DEFs and GEFs, cytopathic effects were only observed in DEFs after blind-replication for 4 passages, and viral copy numbers remained at 10^4.5^–10^6.0^ copies/ml. The result was similar to previous reports that NGPV could be isolated and propagated in duck embryos and DEFs are permissive to NGPV, while GEFs are not [1, 4].

To better characterize SD15, growth cycle, CPE, cell cytotoxicity, and cell apoptosis were investigated in NGPV-infected DEFs. At 0–72 hpi, low levels of green fluorescence were observed in NGPV-infected DEFs, which was regarded as the eclipse period for NGPV. The green fluorescence signal increased rapidly from 72 to 96 hpi, representing the logarithmic phase for SD15. Viral growth plateaued between 96 and 120 hpi with a maximum green fluorescence signal at 96 hpi, representing the best time for viral harvest. By 72 hpi, the cytotoxic effects and CPE of infected DEFs are present.

As a non-enveloped virus, parvovirus releases viral replicates through cell lysis during late infection [18, 19]. This corresponds to NGPV as well. The viral progeny of NGPV was released and then infected the nearby cells, at last causing cytotoxic effects and cell death.

The cytotoxic effect is induced primarily by NS1 [20, 21], which plays an important role in the replication process of parvovirus [22, 23]. NS1 is a multifunctional protein and can also induce apoptosis when parvovirus infects cells [24, 25], but surprisingly, NGPV did not induce apoptosis in our experiments.

Though NGPV and GPV had high similarity, they infected different susceptible hosts. NGPV caused Mule and Cherry ducklings SBDS but no obvious clinical symptom on geese and Muscovy ducks. However, NGPV also had a potential hazard to the waterfowl breeding industry. Previous research by Wentao Fan indicated that the evolutionary rate of NGPV was similar to that of an RNA virus and much faster than those of GPV and MDPV. The recent wild, attenuated GPV diverged from NGPV, indicating that geese may be asymptomatic NGPV carriers [26]. Shifeng Xiao’s research found that NGPV had a wide range of hosts in waterfowl, and NGPV could cause geese and Muscovy ducks to lose weight without significant SBDS [27]. What’s more, ducks coinfected with duck circovirus (DuCV) and NGPV show high coinfection rates, indicating that DuCV may be very important for the disease [28].

## Conclusion

SBDS was caused by NGPV, which has been becoming a threat to the duck-feeding industry of China. In this study, we isolated NGPV strain SD15, and identified its detailed growth characteristics. We found that NGPV can replicate in duck embryos and cause DEF cells death. What’s more, it was very interesting that NGPV cannot induce the apoptosis, which was different from the most parvovirus. Future studies with a focus on vaccine development and identifying the mechanisms of NGPV infection are necessary to address this problem going forward.

## Abbreviations

7-AAD: 7-Amino-Actinomycin
CPE: Cytopathic effects
DEFs: Duck embryo fibroblasts
DEFs: Goose embryo fibroblasts
DuCV: Duck circovirus
GEF: Goose embryo fibroblast
GPV: Goose parvovirus
hpi: Hours post-inoculation
IFA: Indirect fluorescent antibody assay
ITRs: Inverted terminal repeats
MDPV: Muscovy duck parvovirus
NGPV: Novel goose parvovirus
ORFs: Open reading frames
PBS: Phosphate buffered saline
PBS: Phosphate buffered saline
qPCR: Real-time quantitative PCR
SBDS: Short beak and dwarfism syndrome
